# Environmental Factors Controlling the Distribution of *Symbiodinium* Harboured by the Coral *Acropora millepora* on the Great Barrier Reef

**DOI:** 10.1371/journal.pone.0025536

**Published:** 2011-10-31

**Authors:** Timothy F. Cooper, Ray Berkelmans, Karin E. Ulstrup, Scarla Weeks, Ben Radford, Alison M. Jones, Jason Doyle, Marites Canto, Rebecca A. O'Leary, Madeleine J. H. van Oppen

**Affiliations:** 1 Australian Institute of Marine Science, Oceans Institute, University of Western Australia, Crawley, Australia; 2 Australian Institute of Marine Science, Townsville, Australia; 3 DHI Water and Environment, West Perth, Australia; 4 Centre for Spatial Environmental Research and Coral Reef Ecosystems Lab, University of Queensland, St. Lucia, Australia; 5 Centre for Environmental Management, Central Queensland University, Rockhampton, Australia; US Dept. of Agriculture – Agricultural Research Service (USDA-ARS), United States of America

## Abstract

**Background:**

The *Symbiodinium* community associated with scleractinian corals is widely considered to be shaped by seawater temperature, as the coral's upper temperature tolerance is largely contingent on the *Symbiodinium* types harboured. Few studies have challenged this paradigm as knowledge of other environmental drivers on the distribution of *Symbiodinium* is limited. Here, we examine the influence of a range of environmental variables on the distribution of *Symbiodinium* associated with *Acropora millepora* collected from 47 coral reefs spanning 1,400 km on the Great Barrier Reef (GBR), Australia.

**Methodology/Principal Findings:**

The environmental data included Moderate Resolution Imaging Spectroradiometer (MODIS) satellite data at 1 km spatial resolution from which a number of sea surface temperature (SST) and water quality metrics were derived. In addition, the carbonate and mud composition of sediments were incorporated into the analysis along with in situ water quality samples for a subset of locations. Analyses were conducted at three spatio-temporal scales [GBR (regional-scale), Whitsunday Islands (local-scale) and Keppel Islands/Trunk Reef (temporal)] to examine the effects of scale on the distribution patterns. While SST metrics were important drivers of the distribution of *Symbiodinium* types at regional and temporal scales, our results demonstrate that spatial variability in water quality correlates significantly with *Symbiodinium* distribution at local scales. Background levels of *Symbiodinium* types were greatest at turbid inshore locations of the Whitsunday Islands where SST predictors were not as important. This was not the case at regional scales where combinations of mud and carbonate sediment content coupled with SST anomalies and mean summer SST explained 51.3% of the variation in dominant *Symbiodinium* communities.

**Conclusions/Significance:**

Reef corals may respond to global-scale stressors such as climate change through changes in their resident symbiont communities, however, management of local-scale stressors such as altered water quality is also necessary for maintenance of coral-*Symbiodinium* associations.

## Introduction

Unicellular photosynthetic symbionts (*Symbiodinium* spp.) play a vital role in the energy budget, metabolism and secretion of the calcium carbonate skeleton of scleractinian corals [1], [2]. The *Symbiodinium* community associated with scleractinian corals is widely considered to be influenced by host identity and environmental factors and has been shown to shape the coral's tolerance to environmental extremes [3], [4]. *Symbiodinium* is a diverse dinoflagellate genus comprising nine phylogenetic clades (A–I), which are subdivided into numerous types based on ribosomal and chloroplast DNA [5], [6]. Of the nine known clades, six (A–D, F and G) have been identified from scleractinian corals with clades C and D being dominant in the Indo-Pacific. Functional differences among clades and types are known to confer competitive advantages to their host leading to increased resistance to thermal stress (e.g. [4], [7], [8]) and diversification into low light mesophotic habitats [9], [10].

Sea surface temperature (SST) is an important influence on the coral-*Symbiodinium* association under natural conditions [11]–[13] and as a driver of *Symbiodinium* community shifts under bleaching conditions [8] Light is also known to exert important controls on the structure of *Symbiodinium* communities (e.g. [14]). Studies conducted throughout the Caribbean and Indo-Pacific have found that while some clade C types occur abundantly under a variety of thermal and light conditions, clade D is generally found in warmer water or turbid environments [4], [15], [16]. However, the affinity of clade D to certain environments is host-specific and several studies have found clade D types to be more abundant in both shallow, high light environments [10], [13], [17] and low light or turbid environments (Great Barrier Reef, GBR; [18]–[20]).

Studies of the genetic diversity of *Symbiodinium* over large-scale spatial gradients have shown geographically distinct populations that differ with latitude or inshore to offshore conditions (e.g. [15], [21]). The general conclusion from these studies is that *Symbiodinium* diversity is driven by acclimatization to local environments (e.g. latitudinal changes in SST; inshore to offshore conditions). Over smaller spatial scales, variation in *Symbiodinium* community composition across depth gradients [7], [22] has been shown to be greater than over larger horizontal spatial scales [13], further suggesting that light is an important driver of *Symbiodinium* diversity. Thus, local-scale patterns cannot be extrapolated to regional scales given that environmental drivers of coral-*Symbiodinium* associations operate at different spatial scales [23]. In addition to variability in their spatial distributions, *Symbiodinium* associations are flexible over temporal scales with evidence of shuffling from thermo-sensitive to tolerant types in adult corals during SST anomalies on time scales of months and reverting to their post-bleaching consortia within months or several years [8], [24].

The influence of other environmental drivers such as water quality, nutrient levels and sediment-types on *Symbiodinium* biogeography remains poorly understood [25]. Nutrient levels may play a role given their importance in symbiont metabolism and biomass dynamics [26], while sediment type may be important as the free-living stage of *Symbiodinium* associate predominantly with the benthos [27]. A better understanding of the specific environmental drivers of *Symbiodinium* biogeography is fundamental for prediction of coral community responses to a changing climate. To achieve this, comprehensive studies that examine the influence of multiple predictors are required.

The scleractinian coral *Acropora millepora* (Ehrenberg, 1834) has been shown to host a variety of *Symbiodinium* clades and types including C1, C2, A and D (based on ITS1-SCCP or ITS1-QPCR) either individually or simultaneously [4], [8], [19], [20], [28]. Symbiont populations may undergo change [5] in response to transplantation to a different environment [4] or following a natural bleaching event [8]. The flexibility of *A. millepora*-*Symbiodinium* associations makes it an ideal model to test the importance of environmental drivers in shaping the distribution of *Symbiodinium*. This study reports on the correlation between a range of environmental variables and the distribution of *Symbiodinium* associated with *A. millepora* collected at different spatial scales: i) along 13° latitude spanning approximately 1,400 km on the GBR, ii) along a persistent water quality gradient in the Whitsunday Islands spanning approximately 65 km [29], and iii) on populations sampled repeatedly over a number of years at Davies and Trunk Reefs, and the Keppel Islands. The environmental predictors combine satellite data for the regional-scale analysis with water quality samples collected in the Whitsunday Islands for the local-scale analysis. Our results suggest that *Symbiodinium* distribution in *A. millepora* is not always primarily driven by temperature, but that it is dependent on the combination of a variety of environmental variables. Further, depending on the thermal history, the spatial and temporal scales over which a study is conducted are likely to influence the patterns observed.

## Results

**Figure 1:**
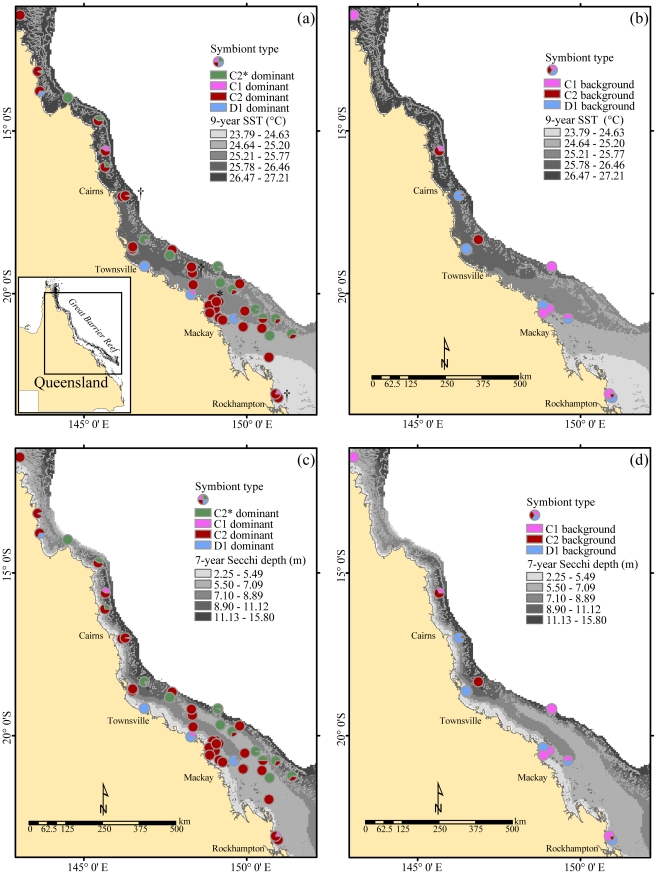
Geographic distribution of *Symbiodinium* types in *A. millepora* on the Great Barrier Reef. Dominant (a) and (c), and background (b) and (d) levels of *Symbiodinium*. Underlay for (a) and (b) is long-term mean SST (9-year; °C) and for (c) and (d) long-term mean Secchi depth (7-year; m). * indicate local-scale data points, † indicate samples included in temporal analysis. Background levels of *Symbiodinium*, determined based on co-occurring lower intensity bands, was detected in only 12% of samples; hence the (b) and (d) represent a smaller sample size.

The most prolific symbiont type that was observed in *A. millepora* was *Symbiodinium* C2, which was dominant in 982 of the 1,527 samples and present at 42 of the 47 reefs investigated (Figure 1a, Table S1). *Symbiodinium* C2* (sensu [4]) was dominant in 274 samples and present at 14 reef locations. *Symbiodinium* C1 was dominant in 69 samples and present at nine reef locations. *Symbiodinium* D1 was dominant in 200 samples and present at 11 reef locations (Figure 1a, Table S1). In the Whitsunday Islands, *Symbiodinium* C2 was the dominant type at all locations sampled. *Symbiodinium* C1 and D1 occurred at background levels at three inner locations only (Repulse, Lindeman and Long Islands) (Table S1).

More than one *Symbiodinium* type was detected in 12% of the samples. There were 83 samples with background levels of *Symbiodinium* C1 at 10 different reef locations (dominant *Symbiodinium* C2 although a sample from Miall Island had D1 as its dominant community in 2006) (Figures 1b and d, Table S1). A further 57 samples had background levels of *Symbiodinium* C2 at eight different reef locations (dominant *Symbiodinium* C1, and to a lesser extent dominant D1), while 42 samples had background levels of *Symbiodinium* D1 at eight different reef locations (dominant *Symbiodinium* C2, and to a lesser extent dominant C1) (Figures 1b and d, Table S1). Finally, two individual samples originating from Whitsunday and Calder Islands hosted background levels of a type belonging to *Symbiodinium* clade A (Figures 1b and d, Table S1).

### Regional-scale patterns, Great Barrier Reef

**Figure 2:**
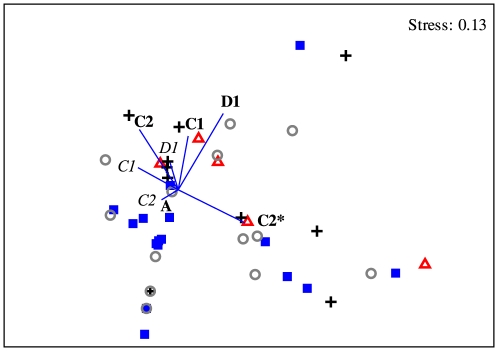
Two-dimensional MDS plot of the *Symbiodinium* community sampled at each of four regions on the Great Barrier Reef. Symbols: ▵ Far Northern, + Northern, ▪ Whitsundays, ○ Southern GBR. For symbiont types, bold indicates a dominant type and italics are those occurring at background levels.

The *Symbiodinium* community differed among regions on the GBR (Table 1) although the pairwise comparisons following a Bonferroni correction were unable to discriminate where those differences occurred. Notwithstanding, a multidimensional scaling (MDS) ordination showed that the *Symbiodinium* communities in regions adjacent to each other at either end of the spatial gradient, i.e. Far Northern and Northern; and Whitsundays and Southern GBR, are more similar to each other and tended to group together (Figure 2).

**Table 1:** Output of the analysis of similarity (ANOSIM) test of *Symbiodinium* communities sampled in each of the four regions (Far Northern, Northern, Whitsundays and Southern GBR) of the Great Barrier Reef.

Comparison	Global R	P
Among regions	0.086	0.043
Pairwise Tests		
Far Northern, Northern	−0.109	0.759
Far Northern, Whitsundays	0.173	0.138
Far Northern, Southern GBR	0.269	0.036
Northern, Whitsundays	0.155	0.030
Northern, Southern GBR	0.176	0.013
Whitsundays, Southern GBR	−0.039	0.831

*Bonferroni correction for multiple comparisons, α = 0.008.*

Results of the marginal tests show that there were several environmental predictors that each explained a significant proportion of the variation in the *Symbiodinium* community, when considered alone (Table 2). The mud and carbonate content of the sediment explained 27.3% and 18.5%, respectively, of the variation in the *Symbiodinium* community while anomalies in Secchi depth (1.8%) had the least influence on *Symbiodinium* communities at regional scales (Table 2). The step-wise model selection eliminated Secchi depth anomalies from the model and the conditional tests associated with the sequential addition of the other predictors were significant for mud content, carbonate content, SST anomaly and mean summer SST (P<0.05; Table 2). Whilst the addition of long-term SST and Secchi depth contributed to the selection of the best model they did not explain significantly more of the variation in the *Symbiodinium* community (Table 2). Together these environmental predictors explained 51.3% of the regional variation in the *Symbiodinium* community on the GBR.

**Table 2:** Summary of regional-scale analyses for model selection to examine the relationship between *Symbiodinium* communities and environmental variables.

Marginal Tests						
Group	Cumulative adjusted R^2^	df	Pseudo-F	P	% Variance explained	% Cumulative variance
Mud			2.56	0.0001	27.3	
Carbonate			2.45	0.0012	18.5	
SST 9 y			5.56	0.0002	10.8	
SST Summer			3.39	0.0060	6.9	
SST anomaly			2.62	0.0270	5.4	
Secchi depth 7 y			1.46	0.1977	3.1	
Secchi depth 3-month anomaly			0.86	0.5219	1.8	

Sequential Tests						
Mud	0.17	7	2.56	0.0001	27.3	27.3
Carbonate	0.24	11	2.01	0.0139	13.0	40.2
SST anomaly	0.27	12	2.48	0.0332	3.9	44.1
SST Summer	0.30	13	2.37	0.0427	3.5	47.6
SST 9 y	0.30	14	1.39	0.2182	2.1	49.7
Secchi depth 7 y	0.31	15	1.12	0.3437	1.6	51.3

*Predictors selected by stepwise selection of terms for the model with the best fit based on Adjusted R^2^.*

**Figure 3:**
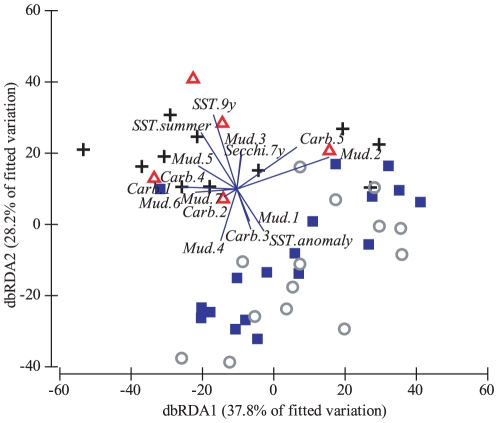
Distance based redundancy ordination (dbRDA) for the fitted model of *Symbiodinium* communities associated with the hard coral *Acropora millepora* and environmental parameters for four regions of the Great Barrier Reef. Symbols: ▵ Far Northern, + Northern, ▪ Whitsundays, ○ Southern GBR.

The distance-based redundancy ordination (dbRDA) indicated that the first two axes explain 66% of the variability of the fitted model (Figure 3). The first axis was related to both the mud and carbonate content of the sediment while the second axis was strongly related to long-term mean SST and represented the spatial structure in the data with the warmer regions, i.e. Far Northern and Northern, located near the top of the plot while the cooler regions were generally situated in the lower half of the plot.

**Figure 4:**
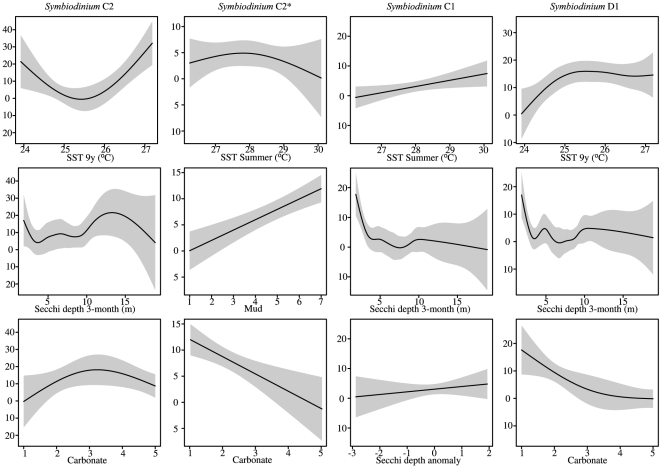
Partial plots of the GAMs of dominant *Symbiodinium* types along gradients of long-term SST (9-year), SST summer mean, 3-month Secchi depth, anomalies in Secchi depth, mud and carbonate content of sediments on the Great Barrier Reef. Solid line shows the model and grey area is the 95% confidence interval. Y-axis is scaled to abundance, values around zero indicate absent symbiont type, negative units are included as they represent the range of confidence limit boundaries.

The distribution of the different *Symbiodinium* types occurring at regional scales on the GBR was influenced by all of the environmental predictors examined (Table 3). The distribution of *Symbiodinium* C2 showed non-linear responses to most of the environmental predictors. For example, *Symbiodinium* C2 occurred in lowest relative abundances at locations with mean summer SST of around 25.5°C and increasing in relative abundance at higher and lower temperatures (Figure 4). Similarly, this symbiont type occurred in greatest relative abundance at locations where the sediments were characterised as having transitional carbonate content, and at locations with a long-term Secchi depth of approximately 13 m (Figure 4).

**Table 3:** Summary of GAMs to examine the environmental drivers associated with the distribution of dominant *Symbiodinium* types sampled at regional scales across the Great Barrier Reef.

	Estimated df	Reference df	F	P
*Symbiodinium* C1				
Secchi 3-month	2.10	2.75	2.69	0.0589
Secchi depth 3-month anomaly	0.68	0.89	2.15	0.1491
SST summer	1.45	2.07	1.43	0.2468
*Symbiodinium* C2				
SST 9 y	3.34	4.16	4.58	0.0026
Secchi 3-month	1.46	1.93	3.99	0.0253
Carbonate	2.73	1.28	2.13	0.0376
*Symbiodinium* C2*				
Mud	2.51	0.65	3.89	0.0003
Carbonate	2.04	0.60	3.37	0.0014
SST summer	3.01	3.91	4.08	0.0059
*Symbiodinium* D1				
Carbonate	5.33	0.95	5.59	<0.0001
SST 9 y	3.88	4.76	3.53	0.0088
Secchi 3-month	2.56	3.25	3.45	0.0202
Mud	1.05	0.46	2.28	0.0265

There was a significant relationship between the distribution of *Symbiodinium* C2* and mean summer temperature. The greatest abundance of *Symbiodinium* C2* occurred at locations where the SST values were around 28°C. In addition, there was a greater abundance of *Symbiodinium* C2* at locations with relatively low carbonate and mud content in the sediments (Figure 4).

The distribution of *Symbiodinium* D1 was strongly influenced by several environmental predictors (Table 3). This symbiont type showed greatest abundance at locations with high carbonate content of the sediment and with the warmest mean summer SST. Additionally, there was a significant relationship between *Symbiodinium* D1 and 3-month Secchi depth. *Symbiodinium* D1 was more abundant at turbid, inner locations with low Secchi depth than at outer locations near the GBR shelf edge (Figure 4).

*Symbiodinium* C1 generally occurred in low relative abundances throughout the GBR. Nevertheless, the relative abundance of this type tended to be greater at turbid, inner locations with lower Secchi depth than at the outer locations near the GBR shelf edge (Figure 4).

### Local-scale patterns, Whitsunday Islands

**Figure 5:**
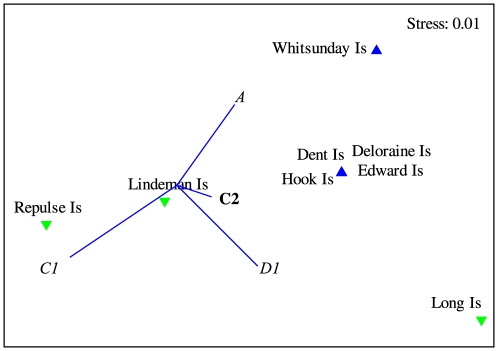
Two-dimensional MDS plot of the *Symbiodinium* community sampled at each of two water quality zones along a gradient in the Whitsunday Islands of the Great Barrier Reef. Symbols: ▾ inner zone, ▴ outer zone. For symbiont types, bold indicates a dominant type and italics are those occurring at background levels. Locations defined as occurring in inner and outer zones of water quality using thresholds described by De'ath and Fabricius (2010) [37].

The *Symbiodinium* community differed between inner and outer zones in the Whitsunday Islands (Table 4) and the MDS plot showed a clear separation of the *Symbiodinium* communities between the two zones (Figure 5). Differences between zones were due to background levels of *Symbiodinium* C1 and D1 at inner locations, which were absent at locations in the outer zone. These two *Symbiodinium* types together accounted for over 92% of the dissimilarity between zones in the Whitsunday Islands (Table 4).

**Table 4:** Summary of multivariate analyses using ANOSIM and SIMPER of *Symbiodinium* communities sampled in each of the two zones (inner and outer) along a water quality gradient in the Whitsunday Islands.

ANOSIM		
Comparison	Global R	P
Between zones	0.703	0.018

SIMPER		
Type	Average dissimilarity	% Cumulative dissimilarity
*Symbiodinium* C1 background	18.29	63.66
*Symbiodinium* D1 background	8.24	92.33

Results of the marginal tests show that there were several environmental predictors that individually explained a significant proportion of the variation in the *Symbiodinium* community (Table 5). The mud content of the sediment, long-term SST and the water quality index (WQI) each explained 80.3%, 39.1% and 37.6% of the variation in the *Symbiodinium* community, respectively whereas anomalies in Secchi depth (2.5%) had the least influence (Table 5). The step-wise model selection eliminated three of the predictors from the model and the conditional tests associated with the sequential addition was significant for mud content and long-term SST (P<0.05; Table 5). Although the addition of long-term SST, mean summer SST and WQI contributed to the selection of the best model they did not explain significantly more of the variation in the *Symbiodinium* community. Together these environmental predictors explained 99.9% of the local-scale variation in the *Symbiodinium* community in the Whitsunday Islands.

**Table 5:** Summary of local-scale analyses for model selection to examine the relationship between *Symbiodinium* communities and environmental variables.

Marginal Tests						
Group	Cumulative adjusted R^2^	df	Pseudo-F	P	% Variance explained	% Cumulative variance
Mud			5.44	0.0368	80.3	
SST 9 y			3.85	0.0363	39.1	
WQI			3.61	0.0516	37.6	
Secchi depth 3-month			2.91	0.0941	32.6	
Secchi depth 7 y			2.48	0.1301	29.2	
Carbonate			0.79	0.6250	24.0	
SST anomaly			0.93	0.4205	13.4	
SST Summer			0.89	0.4529	12.9	
Secchi depth 3-month anomaly			0.15	0.8331	2.5	

Sequential Tests						
Mud	0.66	4	5.44	0.0361	80.3	80.3
SST.9 y	0.81	3	4.33	0.1114	11.6	91.9
SST Summer	0.95	2	9.96	0.1098	6.7	98.7
WQI	1.00	1	33.13	0.1098	1.3	99.9

*Predictors selected by stepwise selection of terms for the model with the best fit based on adjusted R^2^.*

**Figure 6:**
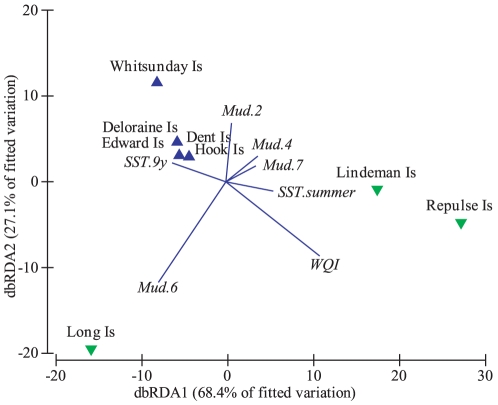
dbRDA ordination for the fitted model of *Symbiodinium* communities associated with the hard coral *Acropora millepora* and environmental parameters for two zones of water quality in the Whitsunday Islands. Symbols: ▾ inner zone, ▴ outer zone.

The output of the model selection of the dbRDA ordination indicates that the first two axes explained over 95% of the variability of the fitted model (Figure 6). Indeed, the first axis was strongly and positively related to the WQI and negatively related to sediment with dominant mud (60–80%).

In the Whitsunday Islands, *Symbiodinium* C2 was the dominant type at all locations sampled. Although long-term Secchi depth was the best single predictor model of the environmental variables that were examined, the analyses did not identify any significant environmental predictors of *Symbiodinium* C2 distribution (Table 6). This was not the case for background levels of *Symbiodinium* where the WQI had a significant influence on their distribution in the Whitsunday Islands (Table 6). The background levels of *Symbiodinium* occurred in greatest relative abundance at turbid locations with high WQI, which corresponded to the inner Whitsunday Islands of Repulse, Lindeman and Long Islands.

**Table 6:** Summary of GAMs to examine the environmental drivers associated with the distribution of the dominant *Symbiodinium* type sampled in the Whitsunday Islands.

	Estimated df	Reference df	F	P
*Symbiodinium* C2				
Secchi depth 7 y	3.298	3.690	2.853	0.1780
*Symbiodinium* background				
Water Quality Index	3.845	3.984	82.42	0.0020

### Temporal changes in distribution of *Symbiodinium*

Shifts in the *Symbiodinium* community occurred at four of the six locations sampled as part of the temporal analysis (Table 7). Trunk Reef was sampled in February 2005 and in March 2009. In 2005, *Symbiodinium* C1 was dominant but changed to *Symbiodinium* C2* as the dominant type in 2009 (Table S2). North Keppel Island was sampled on five occasions between 2001 and 2009. *Symbiodinium* C2 was dominant in all colonies sampled in 2001, but changed to a mix of C2 and D1 in July 2002 following a bleaching event in early 2002, followed by a mix of C1, C2 and D in October 2003 reverting back to 100% *Symbiodinium* C2 dominance by 2009 (Table S2). Miall Island was sampled on three occasions in 2004, 2006 and 2008. In 2004, *Symbiodinium* C2 was the dominant type but there was a shift to *Symbiodinium* D1 and mixtures of C1, C2 and D1 after a bleaching in 2006 followed by a change back to predominantly *Symbiodinium* C2 in 2008 (Table S2). Halfway Island was sampled on seven occasions between 2002 and 2006. *Symbiodinium* C2 was dominant in all years at this location except 2006 when *Symbiodinium* D1 was dominant in 40% of samples (Table S2). There were no changes in the assemblage at either Davies Reef or Magnetic Island where the dominant types were *Symbiodinium* C2* and D1, respectively, over the time period examined.

**Table 7:** Results of Pearson's Chi-square test comparing temporal changes in *Symbiodinium* assemblages at each location.

Location	Chi-squared	df	P
Trunk Reef	76.97	3	<0.0001
North Keppel Island	395.75	20	<0.0001
Miall Island	89.18	12	<0.0001
Halfway Island	341.52	36	<0.0001

**Figure 7:**
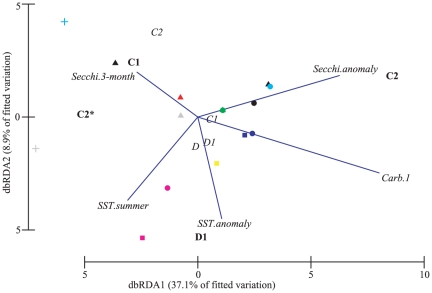
dbRDA ordination for the fitted model of *Symbiodinium* communities associated with the hard coral *Acropora millepora* and environmental parameters for different years of sampling at Trunk Reef and the Keppel Islands. Symbols: + Trunk Reef, ▴ North Keppel Is., ▪ Miall Is., • Halfway Is. Colours: red 2001, green 2002, black 2003, navy blue 2004, light blue 2005, pink 2006, yellow 2008, grey 2009. For symbiont types, bold indicates a dominant type and italics are those occurring in background levels.

Each symbiont type associated with the different environmental predictors in contrasting ways over time (Figure 7). As expected, *Symbiodinium* D1 associated strongly with SST anomalies and mean summer SST. However, the other dominant symbiont types associated more strongly with Secchi depth. *Symbiodinium* C1 and C2* were associated with mean Secchi depth over the 3-months prior to sampling while *Symbiodinium* C2 in the Keppel Islands were associated with anomalies in Secchi depth (Figure 7).

## Discussion

One of the fundamental gaps underlying studies of *Symbiodinium* biogeography is the lack of highly resolved spatial and temporal biological and environmental data. A combination of meta-analysis and field data for coral samples, coupled with large-scale satellite data as well as in situ water quality and sediment data provided a high resolution spatio-temporal dataset for this study. This has deepened our understanding of environmental controls of the patterns of *Symbiodinium* diversity and distribution in *A. millepora* by establishing that factors other than SST influence *Symbiodinium* biogeography and local- and regional-scale patterns are influenced by a variety of factors.

### Regional-scale drivers of symbiont communities

Our results show that spatial and temporal scales are important for determining the environmental drivers that have the strongest influence on *Symbiodinium* associations in *A. millepora* on the GBR. At regional scales, the patterns of variability in *Symbiodinium* distribution were best explained by a combination of mud and carbonate content as well as SST anomalies and mean summer SST. Sediment types can influence the level of resuspension in the water column and therefore light levels but may also act as a reservoir of *Symbiodinium* for uptake by corals such as *A. millepora* that acquire new symbionts from their environment every generation. It is unknown how sediments influence the symbiont community in corals, but since the free-living stage of *Symbiodinium* is predominantly sediment-associated [27], it is conceivable that sediment type and particle size could influence the ecology of the free-living symbionts. While the spatial distribution of mud and carbonate broadly co-vary with distance from shore and water clarity (as a proxy for light), our data show that water clarity per se (here measured as Secchi depth) correlates weakly with symbiont type suggesting that factors other than distance from shore are implicated.

Anomalies in SST and mean summer SST that exceed the thermal tolerance of corals are conducive to bleaching conditions and may drive the shuffling of *Symbiodinium* types as observed by [4] and [8] for *A. millepora*. The GBR-wide distribution of the most abundant symbiont type, *Symbiodinium* C2, appears to be driven by long-term SST, mean summer SST as well as long-term and 3-month Secchi depth and mud content of the sediment, suggesting that it is adapted to a broad range of mean, non-anomalous, temperature regimes and water quality conditions. The fact that the remaining *Symbiodinium* types associated strongly with the optical properties of the water column and sediment composition suggests that water clarity and possibly nutrient levels may play a role in less abundant *Symbiodinium* consortia, with anomalous thermal regimes an important co-driver.

The distribution of *Symbiodinium* D1 was strongly related to many of the environmental predictors that were examined. Previous studies have focused predominantly on temperature and have found a strong association between temperature extremes and D1 prevalence [16], [23]. However, our results suggest that water clarity and sediment type may also be important factors governing the distribution of D1. Although the association of D1 with water clarity is generally consistent with the conclusion of LaJeunesse et al. (2010) [15], we suggest that caution be applied in interpreting results where the standard chlorophyll-*a* concentration algorithm [30] is used as a measure of water clarity in shallow coral reef waters, especially at low (e.g. 24 sq km) resolution. The empirical chlorophyll-*a* concentration algorithm was developed for open ocean Case-1 waters [31] and is unable to correct for bottom reflectance or the presence of scattering particles resulting in significant contamination of the signal in shallow or turbid (Case 2) waters.

*Symbiodinium* clade D is pandemic but uncommon on a global-scale, occurring predominantly on reefs that are subjected to periodic stress or that have a history of bleaching. As such, clade D types have been described as opportunistic endosymbionts that are able to out-compete other types in health-compromised corals [32]. This view is inconsistent with the observation of stable D1 symbiont communities in *A. millepora* at locations such as Magnetic Island, which is difficult to classify as “health-compromised” in the context of coral cover, juvenile coral recruitment, or coral species diversity [33]. Here, D1 prevalence has been shown to be governed by a combination of environmental predictors, hence it is unlikely that stress conditions alone drive their abundance. Rather, responses to environmental conditions coupled with possible local adaptation operate in concert to fulfil their physiological requirements [5], [23], [32].

### Influence of local-scale variation in environmental drivers (water quality) on *Symbiodinium* distribution

Aside from SST and light levels, environmental variables such as water quality have generally not been considered previously as environmental controls on the distribution of *Symbiodinium*, despite having known effects on symbiont density (e.g. [26], [34]) and photo-physiology [35], [36]. The environmental gradients observed across the Whitsunday Island region are stronger and more persistent than those observed along the GBR [29], [37] and have been linked to changes in photo-physiology and coral assemblages with increasing distance away from the discharge of two local rivers [36], [38], [39]. The pattern of variability of *Symbiodinium* types was best explained by the mud content of sediment, the gradient of which is particularly strong in the Whitsunday Islands. Our local-scale results also show that the distribution of *Symbiodinium* C2 was best explained by long-term Secchi depth, highlighting again that water quality, and possibly nutrient levels, are important drivers of *Symbiodinium* distribution. Further, Secchi depth has previously been shown to correlate with large-scale differences in Mesoamerican symbiont communities [40]. As such, water clarity can exert strong local structuring on the marine environment due to terrestrial runoff [41], and wind driven resuspension of sediments [42], that in turn can drive changes at all trophic levels on coral reefs.

It is unclear why Secchi depth related more strongly to *Symbiodinium* C2 at a local-scale compared to the regional GBR-scale. It may simply be a function of higher local-scale sampling intensity of the environmental gradients, which could produce stronger statistical associations. On the other hand, Oliver and Palumbi (2009) [23] also found that regional-scale thermal correlates could not be demonstrated in the west Pacific. They attributed their findings to other factors such as host responses, other environmental drivers, or within-type physiological diversity. Given the high potential for local adaptation of *Symbiodinium* as a result of large population sizes, significant existing heritable genetic variation in physiological performance [43], [44], and restricted gene flow among GBR populations [45], it should not be surprising that responses differ between regional and local scales. The possibility that local-scale environmental conditions may structure *Symbiodinium* associations in contrasting ways to those observed at larger spatial scales warrants further investigation.

### Temporal variability & acclimatization

The occurrence of temporally variable symbiont communities at some locations and not at others is an issue that remains poorly understood. Three of the four sites where substantial temporal changes took place occurred in the Keppel Island group. Symbiont shuffling in this area has been documented following a natural bleaching event in early 2006 [8] but has clearly also taken place previously as a result of bleaching in 2002 [46]. Similarly, *A. millepora* at Trunk Reef in the central GBR underwent a shift from *Symbiodinium* C1 towards the end of a warm summer in 2005 to *Symbiodinium* C2 in 2009. This is the first documented case of temporal symbiont variability on an offshore reef on the GBR indicating that shuffling is not restricted to the more environmentally variable inshore reefs. It is worth noting that all of the temporal changes in symbiont communities presented can be directly correlated to thermal stress (Keppels: 2002 and 2006 bleaching events; Trunk Reef: 2005). This highlights the importance of thermal stress as the driver of symbiont change and the need for standardizing data to non-stressful periods prior to undertaking large-scale analyses to avoid extra sources of variation. The stronger association of *Symbiodinium* C1 with Secchi depth than with temperature is probably an artefact of the data since it is influenced substantially by a successional change in symbiont types at North Keppel and Miall Islands after bleaching. At these sites symbiont communities changed from *Symbiodinium* C2 to *Symbiodinium* D1 and C1 dominance during and after bleaching, and remained as a mix of predominantly *Symbiodinium* C1 and *Symbiodinium* D1 over following months, eventually drifting back to C2 dominance over a period of 1–2 years. *Symbiodinium* C1 is considered thermo-tolerant [47] with a photo-physiology that allows it to out-compete *Symbiodinium* D1 over a period of time until it is itself out-competed by *Symbiodinium* C2 [47]–[49]. Conversely, symbiont communities remained stable during, and following, the 2002 bleaching event at Magnetic Island (100% D1 dominant) and Davies Reef (100% C2* dominant). These results highlight our limited understanding, not only of the factor(s) that influence symbiont shuffling, but also of the prerequisite symbiont makeup and densities that are required for shuffling to take place.

In summary, given the importance of *Symbiodinium* for holobiont metabolism and health, understanding the environmental controls on the distribution of symbiont types is crucial if we are to manage the resilience of coral reefs in an era of rapid environmental change. Modelling of *Symbiodinium* community structure as well as accumulation of large-scale data in space and time through meta-analysis and satellite imagery is important to allow reliable predictions of biological responses to environmental changes. Our findings highlight that drivers of specific associations between *Symbiodinium* and *A. millepora* are multiple and varied depending on the spatial and temporal scale at which investigations took place. At larger scales, SST variables are important drivers whereas local-scale patterns can be explained by variables that are affected by environmental gradients caused by local events. Importantly, this study is unique among other large-scale studies in that the samples all come from the same coral species, which avoids possible confounding of patterns due to host identity, thus reducing the risk of spurious environmental correlations.

## Materials and Methods

### Sampling design

Sampling of *A. millepora* was divided into two studies based on spatial scales. The first component focused on a regional scale incorporating 47 inner and outer coral reefs across 13° latitude (11°–24°S) of the GBR (Figure 1). In total, dominant and background levels of *Symbiodinium* in 1,527 coral samples were analysed with sampling undertaken between 2001 and 2009 and within-reef replication of 1–79 colonies (mode = 10). The second component focused on 8 of the 47 coral reefs situated in the Whitsunday Islands region (20°00′–30′S and 148°45′–149°15′E) of the GBR. This region is characterised by a persistent environmental gradient with significant changes in irradiance, sediments and water column nutrients from inner to outer locations with increasing distance away from the discharge of two rivers [29]. In total, 79 *Symbiodinium* samples were analysed from eight locations in the Whitsunday Islands with sampling done in January 2007 and within-reef replication of 10 colonies (mode = 10).

To investigate the temporal variability in *Symbiodinium* communities, six locations (Davies Reef, Magnetic Island, Miall Island, North Keppel Island, Trunk Reef, Halfway Island) were sampled repeatedly (n = 2–7 times) between 2001 and 2009 and within-reef replication of 7–79 colonies (mode = 10).

For all collections at each location, branches (∼3–5 cm long) of *A. millepora* were collected from adult colonies occurring at a depth of 1–7 m below lowest astronomical tide (LAT), generally on the leeward side of the reef. *Acropora millepora* generally occurs on the mid- to upper reef slope, including the reef flat on inshore sites, but not on offshore sites where its close congener, *A. spathulata*, is more common. To avoid biasing our collections with respect to water clarity, collections deliberately covered the full depth range of *A. millepora* with collections at 32% of sites including a minimum depth of >2 m and 62% with a maximum depth of >4 m.

### Genetic analysis

**Figure 8:**
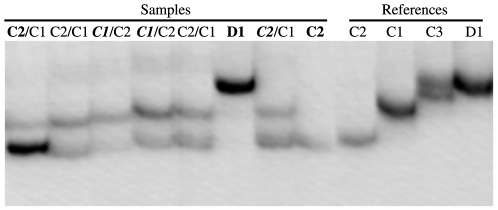
SSCP gel image. The image illustrates the link between band intensity and how relative abundance (dominant or background) of *Symbiodinium* strains was determined for individual samples. Samples in **Bold** = dominant, **Bold**/Normal = dominant/background, Normal/Normal = equal band intensities, ***Bold Italics***/Normal = slight dominance/significant background.

All samples were preserved in absolute ethanol. DNA extraction was carried out using Wayne's method [50] and PCR amplification was conducted following [19]. Genetic identification of *Symbiodinium* types hosted by *A. millepora* colonies was performed using single-stranded conformation polymorphism (SSCP) of the ITS1 region of the nuclear rDNA [19], which has a lower detection limit of 5–10% relative abundance but does not rule out the presence of symbionts below the detection limits [12]. This technique was used because it allowed us to include the results of a relatively large body of literature of *A. millepora* samples that have been genotyped using SSCP with newly genotyped samples. Samples were identified against known *Symbiodinium* SSCP profiles with dominant and background levels identified based on band intensity on the SSCP gel as per the example in Figure 8. PCR products giving distinct SSCP profiles were cloned and sequenced to confirm genotypes (*Symbiodinium* C1: Gen Bank Accession numbers AF380551, EU189440 –1, EU189444 – 7; *Symbiodinium* C2: AF380552, EU189442 – 3, EU189448 – 9; *Symbiodinium* C2*: AY643495 – AY643498; and *Symbiodinium* D1: EU02479, EU189450 – 5).

### Environmental data

#### SST and Secchi Depth

The environmental data used in the analysis included Moderate Resolution Imaging Spectroradiometer (MODIS) satellite data from which a number of temperature and Secchi depth (as a measure of water quality) metrics were derived. Daily MODIS data were acquired directly from the NASA Ocean Biology Processing Group (oceancolor.gsfc.nasa.gov) for the period 2000 to 2009 and various time series generated at 1 km spatial resolution. The SST metrics included the long-term (9-year) mean SST, mean summer SST (01-Dec to 28-Feb) and summer SST anomalies, which were determined as the difference between the previous 3-month summer mean and the long-term summer mean. Both day- and night-time SST data were used.

Recent developments in remote sensing application include the development of an operational algorithm to determine the euphotic zone depth (Zeu_%_), a direct measure of water clarity [51]. The quasi-analytical (QAA) algorithm is based on the inherent optical properties of the water column, a function of absorption and backscattering [51], to determine Zeu_%_ in eutrophic coastal and complex waters. For this study, we used a GBR-validated Secchi depth algorithm, generated by matching the 10% euphotic depth level (Zeu10_%_) against GBR Secchi data (1997–2010) as follows: MODIS and SeaWiFS Level-1 (oceancolor.gsfc.nasa.gov) satellite/in situ data matchups were rigorously selected to ensure optimal accuracy. The matched in situ Secchi data were further regressed against the QAA 10% light level (Zeu_10%_) algorithm and a Type II linear regression (RMA) of log-transformed satellite and in situ data used to fine-tune the Secchi depth (Zsd) returned from the QAA algorithm, to generate a GBR-validated Zsd algorithm [52]. The algorithm was implemented and applied to the full regional time series of MODIS Aqua data (2002–2010). Daily, monthly and climatological means were generated at 1 km resolution for further analyses.

The Secchi depth metrics included the long-term (7-year; 2002–2009) mean Secchi depth, mean Secchi depth for 3 months prior to sampling date, and Secchi depth anomalies. The Secchi depth anomalies were determined as the difference between the means of the 3-months prior to coral sampling and the 3-month long-term means (2002–2009) for that same period. The long-term means were calculated for the 3-month periods over the 7-year Secchi depth data series.

To maintain consistency between data points, Secchi depth data were extracted from the same locations used for extracting the SST values. However, to avoid any potential bottom contamination, particularly at the inshore locations, we compared the physical depth at each location with the 7-year long-term Secchi mean. Where the physical depth was shallower than the long-term mean Secchi depth, an alternative pixel location was manually selected by adjusting the station location to the closest pixel with a physical depth that exceeded the 7-year long-term Secchi mean.

#### Sediments

Mud and carbonate sediment data were obtained from [53]. Carbonate content was derived by measurement of the amount of acid-soluble material and divided in the following categories: i) Pure carbonate facies (>90%), ii) High carbonate facies (80–90%), iii) Impure carbonate facies (60–80%), iv) Transitional facies (40–60%), v) Terrigenous facies (20–40%), and vi) High terrigenous facies (<20%). Terrigenous mud content is divided into the following categories based on the content of mud: i) Pure mud (>80%), ii) Dominant mud (60–80%, iii) Very high mud (40–60%), iv) High mud (20–40%), v) Moderate mud (10–20%), vi) Low mud (1–10%), and vii) Non-mud facies, composed predominantly of sand (<1%).

#### Water quality

Data were collected for thirteen irradiance and water-column nutrient variables at each of the coral sampling locations in the Whitsunday Islands during water quality sampling undertaken from August 2004 to January 2007 [29]. The irradiance measurements included Secchi- and optical depth, while surface water was collected for measurements of chlorophyll *a*, phaeophytin, particulate nitrogen (PN), particulate phosphorus (PP), particulate organic carbon (POC), dissolved inorganic nitrogen (DIN: NH_4_, NO_2_, NO_3_), dissolved inorganic phosphorus (DIP: PO_4_), total dissolved nutrients (total dissolved nitrogen [TDN], total dissolved phosphorus [TDP]) and dissolved organic nutrients (dissolved organic nitrogen [DON] and dissolved organic phosphorus [DOP]). Analysis of the water samples followed standard analytical procedures described in [29]. Since many of these variables are known to be highly correlated with each other, a water quality index (WQI; further details see [36], [54]) was calculated for use as an environmental predictor in the analyses using the sum of a z-score transformation for the irradiance and water-column nutrient variables.

### Statistical analyses

Differences in communities of *Symbiodinium* among regions on the GBR (regional-scale study) and along a persistent water quality gradient in the Whitsunday Islands (local-scale study) were examined with multivariate procedures. For each study, spatial variation among communities of *Symbiodinium* was examined graphically using multidimensional scaling (MDS) ordination. The adequacy of the two-dimensional representation was assessed by examining the stress value. Stress <0.1 indicates that the ordination has accurately represented the relationships among the samples, but values greater than 0.25 indicate that the ordination may have misrepresented the data [55].Variation in *Symbiodinium* communities among four regions on the GBR (Far Northern, Northern, Whitsundays and Southern GBR; regional-scale study) and between inner and outer zones of the Whitsunday Islands (local-scale study) were analysed with one-way analyses of similarities (ANOSIM) using the Bray-Curtis similarity measure. Pairwise comparisons were used to test for differences between pairs of samples when ANOSIM was significant and a Bonferroni correction was used to control the probability of Type I error. Where differences in *Symbiodinium* communities were detected, the type contributing the most to the dissimilarity was identified using similarity of percentages (SIMPER) analyses. The zones of water quality were based on the thresholds described by [37]; inner locations with levels of water column chlorophyll-*a* >0.45 µg l^−1^, outer locations with <0.45 µg l^−1^. The MDS and ANOSIM tests were done using dominant and background levels of *Symbiodinium*, each specified separately, in PRIMER6 with PERMANOVA+, as described by [56], [57].

Modelling of the relationship between the *Symbiodinium* community and the environmental predictor variables was done using distance-based redundancy analysis (dbRDA) following techniques described by [58], [59]. The importance of each environmental predictor was first assessed individually in marginal tests. A stepwise procedure with variable selection based on adjusted R^2^ was then used to identify the model of environmental predictors that best explained the dissimilarity among *Symbiodinium* types in their patterns of abundance. The predictors were fitted sequentially by either adding or subtracting from the model and the results presented are sequential tests for the inclusion of each successive predictor based on the selection of those preceding them in the output. Co-linearity among the environmental predictors was tested prior to analysis and variables that were highly correlated were omitted from the analysis. The sediment data (categorical) were treated as binary in the environmental dataset. The dbRDAs were done using DISTLM in PRIMER6 with PERMANOVA+, as described by [56], [57].

The influence of the environmental predictors on the distribution of the dominant *Symbiodinium* types (e.g. C1, C2, C2* and D1) was then examined using generalized additive models (GAMs). Given the low number of samples where background levels of *Symbiodinium* were detected, individual analyses for each background type was not possible and hence the analyses focused on dominant *Symbiodinium* types and a pooled background type.

For the temporal component of the study, a Pearson Chi-squared test was used to determine shuffling between dominant *Symbiodinium* types at six locations (Magnetic, Halfway, Miall and North Keppel Islands, and Davies and Trunk Reefs) where sufficient samples had been collected on multiple occasions through time (2002–2006). The GAMs and temporal analyses were done using the statistical package R [60].

## Supporting Information

Table S1 **Summary of sampling locations and date,** ***Symbiodinium*** **types, number of replicates and source of data.**
(DOCX)

Table S2 **Summary of sampling locations and date as well as abundance of** ***Symbiodinium*** **types used in the temporal analysis.**
(DOCX)
