# Supplementary material for: Environmental Factors Controlling the Distribution of Symbiodinium Harboured by the Coral Acropora millepora on the Great Barrier Reef
Source: PLoS One. 2011 Oct 31;6(10):e25536. doi: 10.1371/journal.pone.0025536 (PMC3204971; doi:10.1371/journal.pone.0025536)
Supplement: Table S1 — Summary of sampling locations and date, Symbiodinium types, number of replicates and source of data. (DOCX) [file pone.0025536.s001.docx]

Table S1. Summary of sampling locations and date, *Symbiodinium* types, number of replicates and source of data.

| Location | Date | Latitude | Longitude | n | *Symbiodinium* type | Source |
| --- | --- | --- | --- | --- | --- | --- |
| Wallace Is. | Nov-08 | 11.44 | 143.03 | 61 | **C2**, *C1* | This study |
| Night Is. | Nov-08 | 13.17 | 143.58 | 54 | **C1**, **C2**, **D1**, **A** | This study |
| Wilke Is. | Nov-08 | 13.78 | 143.64 | 44 | **C2**, **D1** | This study |
| Tydeman Is. | Nov-08 | 13.98 | 144.50 | 2 | **C2*** | This study |
| Lizard Is. | Nov-08 | 14.69 | 145.44 | 48 | **C2**, **C2*** | This study |
| Emily Reef | Feb-05 | 15.61 | 145.66 | 45 | **C1**, **C2**, *C1, C2* | This study |
| Undine Reef | Nov-08 | 16.11 | 145.65 | 46 | **C2**, **C2*** | This study |
| Sudbury Reef 2 | Feb-05 | 17.00 | 146.27 | 47 | **C1**, **C2**, **D1**, *C2, D1* | This study |
| Sudbury Reef 1 | Feb-05 | 17.02 | 146.17 | 49 | **C2** | This study |
| †Trunk Reef | Feb-05 | 18.34 | 146.86 | 5 | **C1**, *C2* | This study |
| †Trunk Reef | Mar-09 | 18.34 | 146.86 | 16 | **C1**, **C2**, **C2***, **D1** | This study |
| Pelorus Is. | Nov-04 | 18.56 | 146.50 | 49 | **C2** | This study |
| Orpheus Is. | Nov-04 | 18.63 | 146.49 | 50 | **C2**, *D1* | This study |
| Fantome Is. | Dec-04 | 18.66 | 146.51 | 49 | **C2**, *D1* | This study |
| Chicken Reef | Aug-02 | 18.66 | 147.71 | 3 | **C2** | [[20](#_ENREF_20)] |
| †Davies Reef | Feb-02 | 18.82 | 147.63 | 22 | **C2*** | [[4](#_ENREF_4)] |
| †Davies Reef | Sep-02 | 18.83 | 147.64 | 10 | **C2*** | This study |
| †Davies Reef | Apr-05 | 18.83 | 147.64 | 31 | **C2*** | This study |
| †Davies Reef | Jul-01 | 18.83 | 147.64 | 14 | **C2*** | This study |
| †Magnetic Is. | Jun-02 | 19.16 | 146.87 | 10 | **D1** | This study |
| †Magnetic Is. | Apr-03 | 19.16 | 146.87 | 18 | **D1** | This study |
| †Magnetic Is. | Jul-01 | 19.16 | 146.87 | 8 | **D1** | This study |
| Nelly Bay | Jul-01 | 19.16 | 146.85 | 17 | **D1** | [[4](#_ENREF_4)] |
| Darley Reef | Apr-05 | 19.17 | 149.12 | 44 | **C2**, **C2***, *C1* | This study |
| Dingo Reef | Aug-02 | 19.19 | 148.30 | 1 | **C2** | [[20](#_ENREF_20)] |
| Charity Reef | Aug-02 | 19.37 | 148.33 | 18 | **C2**, **A** | [[20](#_ENREF_20)] |
| Line Reef | Mar-05 | 19.67 | 149.19 | 4 | **C2*** | This study |
| UN 19-165 | Aug-02 | 19.70 | 149.79 | 2 | **C2** | [[20](#_ENREF_20)] |
| Holbourne Is. | Oct-03 | 19.73 | 148.35 | 39 | **C2** | This study |
| Ross Reef | Mar-05 | 19.88 | 149.56 | 27 | **C2**, **C2***, **D1** | This study |
| Stone Is. | Aug-02 | 20.03 | 148.29 | 10 | **C1**, **D1** | [[20](#_ENREF_20)] |
| *Deloraine Is. | Jan-07 | 20.16 | 149.07 | 10 | **C2** | This study |
| *Hook Is. | Jan-07 | 20.16 | 148.96 | 9 | **C2** | This study |
| *Edward Is. | Jan-07 | 20.24 | 149.15 | 10 | **C2** | This study |
| *Whitsunday Is. | Jan-07 | 20.25 | 149.07 | 10 | **C2**, *A* | This study |
| *Dent Is. | Jan-07 | 20.35 | 148.92 | 10 | **C2** | This study |
| *Long Is. | Jan-07 | 20.37 | 148.85 | 10 | **C2**, *D1* | This study |
| *Lindeman Is. | Jan-07 | 20.46 | 149.03 | 10 | **C2**, *C1* | This study |
| Boulton Reef | Mar-05 | 20.48 | 150.28 | 34 | **C2**, **C2*** | This study |
| Credlin Reef | Aug-02 | 20.54 | 149.94 | 11 | **C2** | [[20](#_ENREF_20)] |
| *Repulse Is. | Jan-07 | 20.59 | 148.87 | 10 | **C2**, *C1* | This study |
| Allonby Is. | Aug-02 | 20.75 | 149.16 | 7 | **C2** | [[20](#_ENREF_20)] |
| Goble Reef | Mar-05 | 20.76 | 150.50 | 30 | **C2**, **C2*** | This study |
| Calder Is. | Oct-03 | 20.77 | 149.62 | 31 | **C1**, **C2**, **C2***, **D1**, *C1, D1, A* | This study |
| 20-344 Reef | Mar-05 | 20.77 | 150.89 | 14 | C2, C2* | This study |
| Brampton Is. | Aug-02 | 20.81 | 149.26 | 3 | C2 | [[20](#_ENREF_20)] |
| Penrith Is. | Oct-03 | 21.02 | 149.89 | 2 | C2 | This study |
| UN 21-056 | Aug-02 | 21.07 | 150.47 | 3 | C2 | [[20](#_ENREF_20)] |
| 21-121 Reef | Mar-05 | 21.24 | 151.39 | 31 | C2, C2*, D1 | This study |
| Paul Reef | Mar-05 | 21.29 | 150.70 | 7 | C2* | This study |
| High Peak Is. | Oct-03 | 21.96 | 150.68 | 34 | C2 | This study |
| †Nth Keppel Is. | Oct-03 | 23.09 | 150.89 | 37 | **C1**, **C2**, **D1**, *C1, C2* | This study |
| †Nth Keppel Is. | Apr-09 | 23.09 | 150.89 | 7 | **C1**, **C2**, *C1, C2, D1* | This study |
| †Nth Keppel Is. | Jul-02 | 23.09 | 150.89 | 10 | **C2**, **D1**, *C2* | This study |
| †Nth Keppel Is. | Feb-03 | 23.09 | 150.89 | 20 | **C2**, **D1**, *C2* | This study |
| †Nth Keppel Is. | Jul-01 | 23.09 | 150.89 | 28 | **C1**, **C2**, *C1, C2* | This study |
| †Miall Is. | Aug-06 | 23.15 | 150.90 | 79 | **C1**, **C2**, **D1**, *C1, C2, D1, D* | This study |
| †Miall Is. | Dec-04 | 23.15 | 150.90 | 79 | **C2**, **D1**, D1 | This study |
| †Miall Is. | Apr-08 | 23.15 | 150.90 | 14 | **C2**, **D1**, *C1, D1, D* | This study |
| †Halfway Is. | Feb-02 | 23.20 | 150.97 | 20 | **C2, D1**, *C2, D1* | This study |
| †Halfway Is. | Jul-02 | 23.20 | 150.97 | 10 | **C2**, **D1**, *C2* | This study |
| †Halfway Is. | Mar-04 | 23.20 | 150.97 | 18 | **C2**, *D1* | This study |
| †Halfway Is. | Sep-04 | 23.20 | 150.97 | 15 | **C2**, **D1** | This study |
| †Halfway Is. | Aug-06 | 23.20 | 150.97 | 15 | **C1**, **C2**, **D1**, *C1, D1, D* | This study |
| †Halfway Is. | Mar-03 | 23.20 | 150.97 | 20 | **C2**, **D1**, *C2, D1* | This study |
| †Halfway Is. | Mar-05 | 23.20 | 150.97 | 18 | **C2**, *D1* | This study |

Asterisks indicate local-scale data points, † indicate samples included in temporal analysis. For Symbiodinium type, bold indicates a dominant type and italics are those occurring in background levels.
