# Supplementary material for: Environmental Factors Controlling the Distribution of Symbiodinium Harboured by the Coral Acropora millepora on the Great Barrier Reef
Source: PLoS One. 2011 Oct 31;6(10):e25536. doi: 10.1371/journal.pone.0025536 (PMC3204971; doi:10.1371/journal.pone.0025536)
Supplement: Table S2 — Summary of sampling locations and date as well as abundance of Symbiodinium types used in the temporal analysis. (DOCX) [file pone.0025536.s002.docx]

Table S2. Summary of sampling locations and date of *Symbiodinium* types used in the temporal analysis.

|  |  |  | *Symbiodinium* type dominant | | | |  | *Symbiodinium* type background | | | |
| --- | --- | --- | --- | --- | --- | --- | --- | --- | --- | --- | --- |
| Location | Date | n | C1 | C2 | C2* | D1 |  | C1 | C2 | D1 | D |
| Trunk Reef | Feb-05 | 5 | 5 | 0 | 0 | 0 |  | 0 | 5 | 0 | 0 |
| Trunk Reef | Mar-09 | 16 | 1 | 1 | 13 | 1 |  | 0 | 0 | 0 | 0 |
| North Keppel Is. | Jul-01 | 28 | 5 | 23 | 0 | 0 |  | 23 | 5 | 0 | 0 |
| North Keppel Is. | Jul-02 | 10 | 0 | 8 | 0 | 2 |  | 0 | 2 | 0 | 0 |
| North Keppel Is. | Feb-03 | 20 | 0 | 19 | 0 | 1 |  | 0 | 1 | 0 | 0 |
| North Keppel Is. | Oct-03 | 37 | 23 | 12 | 0 | 2 |  | 9 | 18 | 0 | 0 |
| North Keppel Is. | Apr-09 | 7 | 1 | 6 | 0 | 0 |  | 3 | 1 | 4 | 0 |
| Miall Is. | Dec-04 | 79 | 0 | 64 | 0 | 15 |  | 0 | 0 | 6 | 0 |
| Miall Is. | Aug-06 | 79 | 7 | 20 | 0 | 52 |  | 12 | 3 | 5 | 10 |
| Miall Is. | Apr-08 | 14 | 0 | 10 | 0 | 4 |  | 1 | 0 | 1 | 1 |
| Halfway Is. | Feb-02 | 20 | 0 | 18 | 0 | 2 |  | 0 | 1 | 1 | 0 |
| Halfway Is. | Jul-02 | 10 | 0 | 8 | 0 | 2 |  | 0 | 2 | 0 | 0 |
| Halfway Is. | Mar-03 | 20 | 0 | 18 | 0 | 2 |  | 0 | 1 | 1 | 0 |
| Halfway Is. | Mar-04 | 18 | 0 | 18 | 0 | 0 |  | 0 | 0 | 2 | 0 |
| Halfway Is. | Sep-04 | 15 | 0 | 12 | 0 | 3 |  | 0 | 0 | 0 | 0 |
| Halfway Is. | Mar-05 | 18 | 0 | 18 | 0 | 0 |  | 0 | 0 | 2 | 0 |
| Halfway Is. | Aug-06 | 15 | 2 | 7 | 0 | 6 |  | 2 | 0 | 2 | 2 |
